# The connection between aging, cellular senescence and gut microbiome alterations: A comprehensive review

**DOI:** 10.1111/acel.14315

**Published:** 2024-08-15

**Authors:** Dong‐Hyun Jang, Ji‐Won Shin, Eunha Shim, Naoko Ohtani, Ok Hee Jeon

**Affiliations:** ^1^ Department of Biomedical Sciences Korea University College of Medicine Seoul Republic of Korea; ^2^ Department of Pathophysiology Osaka Metropolitan University Graduate School of Medicine Osaka Japan

**Keywords:** aging, cellular senescence, gut microbiome, musculoskeletal diseases, senotherapeutics

## Abstract

The intricate interplay between cellular senescence and alterations in the gut microbiome emerges as a pivotal axis in the aging process, increasingly recognized for its contribution to systemic inflammation, physiological decline, and predisposition to age‐associated diseases. Cellular senescence, characterized by a cessation of cell division in response to various stressors, induces morphological and functional changes within tissues. The complexity and heterogeneity of senescent cells, alongside the secretion of senescence‐associated secretory phenotype, exacerbate the aging process through pro‐inflammatory pathways and influence the microenvironment and immune system. Concurrently, aging‐associated changes in gut microbiome diversity and composition contribute to dysbiosis, further exacerbating systemic inflammation and undermining the integrity of various bodily functions. This review encapsulates the burgeoning research on the reciprocal relationship between cellular senescence and gut dysbiosis, highlighting their collective impact on age‐related musculoskeletal diseases, including osteoporosis, sarcopenia, and osteoarthritis. It also explores the potential of modulating the gut microbiome and targeting cellular senescence as innovative strategies for healthy aging and mitigating the progression of aging‐related conditions. By exploring targeted interventions, including the development of senotherapeutic drugs and probiotic therapies, this review aims to shed light on novel therapeutic avenues. These strategies leverage the connection between cellular senescence and gut microbiome alterations to advance aging research and development of interventions aimed at extending health span and improving the quality of life in the older population.

## Graphical abstract

The review “Cellular senescence and gut microbiome alterations” by J. Dong‐Hyun et al. demonstrates that gut microbiome dysbiosis and cellular senescence interact bidirectionally and contribute significantly to aging. It also highlights possible ways to target this axis to restore intestine homeostasis, which promotes healthy aging and mitigates the effects of age‐related musculoskeletal diseases.

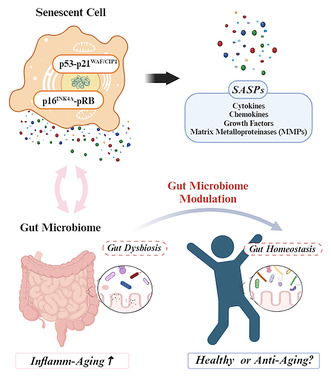

**Abbreviations**
AI: artificial intelligence
ALM: appendicular mean mass
BA: bile acid
BALP: bone–specific alkaline phosphatase
BLM: bleomycin
BMD: bone mineral density
BSH: bile salt hydrolase
CRC: colorectal cancer
CTX: collagen type 1 cross‐linked C‐telopeptide
D: dasatinib
DCA: deoxycholic acid
DKO: double knockout
EGCG: epigallocatechin gallate
FGF: fibroblast growth factor
FMT: fecal microbiota transplantation
FXR: farnesoid X receptor
GC: germinal center
GF: germ‐free
HCC: hepatocellular carcinoma
HSCs: hepatic stellate cells
IBD: inflammatory bowel disease
IECs: intestinal epithelial cells
ILFs: isolated lymphoid follicles
ISCs: intestinal stem cells
LCA: lithocholic acid
LPS: lipopolysaccharide
LS: lumbar spine
MMPs: matrix metalloproteinases
OA: osteoarthritis
PPs: Peyer's patches
PTH: parathyroid hormone
Q: quercetin
ROS: reactive oxygen species
SASPs: senescence‐associated secretory phenotypes
SCAPs: senescent cell antiapoptotic pathways
SCFAs: short chain fatty acids
TLR: Toll‐like receptor
TNF: tumor necrosis factor
Tregs: T regulatory cells
TβMCA: tauro‐beta‐muricholic acid

## 1 INTRODUCTION

Aging is a double‐edged sword: It drives species evolution through generational turnover, promoting genetic diversity and adaptation. Yet, on an individual level, aging is universally regarded as a challenge to be overcome. Despite advancements in nutrition, sanitation, and social welfare contributing to increased life expectancy in developed nations, these gains have not consistently translated into extended periods of good health; thus, they pose significant challenges for sustainable social and healthcare systems. Consequently, research efforts have shifted focus, aiming to extend life expectancy and enhance the quality of life during those extra years, referred to as health span.

Among factors implicated in the aging process in higher animals, two phenomena have recently emerged as critical contributors: The accumulation of senescent cells—cells that have ceased dividing but do not die and are metabolically active—and significant alterations in the composition of the gut microbiota. However, the intricate interplay between these factors and their collective impact on aging has only begun to be unraveled. Emerging evidence suggests that the increase in senescent cells with age, coupled with shifts in gut microbiota composition, exerts a bidirectional influence on the aging process, notably through interactions with the host's immune system. This review lays the groundwork for an in‐depth examination of the relationship between cellular senescence and gut microbiome alterations, highlighting current research findings and identifying critical areas for future exploration.

Building on the foundational work by López‐Otín et al., who outlined nine hallmarks of aging, including genomic instability, telomere attrition, epigenetic alterations, mitochondrial dysfunction, and cellular senescence subsequent updates have proposed expanding this list to include disabled macroautophagy, chronic inflammation, and dysbiosis (López‐Otín et al., 2013, 2023). While extensive research has been conducted on these hallmarks individually, their inter‐relationships and collective impact on aging remain less explored.

This review aims to bridge this gap by delving into the specific roles of cellular senescence and gut microbiome alterations within the broader context of aging. It will explore how these two critical aspects of aging interact with each other and the host immune system, potentially offering new therapeutic targets for mitigating age‐related decline. By examining the state of current research, including the use of senolytic or senomorphic therapies, and highlighting the importance of gut microbiome modulation, this review provides a comprehensive overview of the emerging strategies to promote healthy aging and address age‐associated diseases. Through this exploration, we aspire to highlight the multifaceted nature of aging and the promising avenues for future research and therapeutic intervention.

## 2 INCREASE IN SENESCENT CELLS AND THEIR INFLUENCE ON THE AGING PROCESS

Cellular senescence, a state of permanent cell cycle arrest, was first described in vitro by Leonard Hayflick and Paul Moorhead in 1961. It has since emerged as a key therapeutic target in aging and disease research. Triggered by various stresses, such as DNA damage, telomere shortening, oxidative stress, and epigenetic alterations, cellular senescence is mediated through key pathways such as p16^INK4A^‐pRB and p53–p21^WAF1/CIP1^ (Hayflick, 1965; Rodier & Campisi, 2011). These senescent cells exhibit diverse morphological and functional changes, contributing to tissue and organ function alterations (Hernandez‐Segura et al., 2018). The triggers—ranging from replicative to oncogenic stress, reactive oxygen species (ROS), and proteotoxic stress—induce distinct transcriptomic and proteomic patterns specific to each type of cellular stimulus (Hernandez‐Segura et al., 2017). Moreover, even within the same senescent cells, there is a variability in cell clusters and expressed mRNAs across different stages of senescence (Admasu et al., 2023; Wechter et al., 2023). This “complexity” and “heterogeneity” pose challenges in defining and understanding the aging process.

Another critical feature of senescent cells is the secretion of senescence‐associated secretory phenotypes (SASPs) (Coppé et al., 2010), comprising various cytokines, chemokines, growth factors, proteases, extracellular vesicles, and others (Basisty et al., 2020; Estévez‐Souto et al., 2023; Jeon et al., 2019). SASP factors can induce a pro‐inflammatory environment, leading to gene expression errors and metabolic dysregulation, thereby influencing the aging process and the development of age‐related diseases (Coppé et al., 2011; Hernandez‐Segura et al., 2018; Rodier & Campisi, 2011; Yousefzadeh et al., 2020). The transmission of SASPs to neighboring cells propagates cellular senescence. Interestingly, an aged systemic milieu induces senescence transmission in surrounding tissues, and impair the regenerative capacity of the organisms in young parabionts that received old mice blood (Jeon et al., 2022; Karin & Alon, 2021). Activation of anti‐aging properties present in the blood of young mice or diluting blood plasma in aged mice has been noted to promote rejuvenating effects and improvements in the weakened functions of specific tissues, highlighting the potential benefits of modulating age‐elevated systemic factors (e.g., SASPs) derived from senescent cells for rejuvenation therapies (Kim et al., 2022; Ma et al., 2022; Mehdipour et al., 2021; Mehdipour et al., 2020; Ximerakis et al., 2023; Zhang, Lee, et al., 2023).

The accumulation of senescent cells contributes to “inflammageing”, a state of chronic inflammation that exacerbates age‐related decline in physiological functions and increases the risk of diseases such as osteoporosis, sarcopenia, and osteoarthritis (Coppé et al., 2011; Funk et al., 2023; Gulen et al., 2023; Guo et al., 2022). The immune system's (innate and adaptive immunity) decline, or immunosenescence, further compounds these effects, impairing the body's ability to respond to infections and maintain homeostasis. This gradual deterioration compromises the ability to mount effective immune responses, with aged CD8^+^ T cells, memory B cells, and senescence‐associated macrophages particularly affected (Liu et al., 2023; Ovadya et al., 2018; Yousefzadeh et al., 2021).

Hence, to decrease the rate of inflammageing, far‐reaching research on discovering interventions, precisely targeting and eliminating a specific senescent cell to prevent the accumulation of senescent cells is needed (Moskalev et al., 2022). Research into selectively eliminating senescent cells expressing the p16^INK4a^ gene has shown promise in extending the lifespan and mitigating aging‐related conditions in rodent models, sparking interest in drugs that target senescent cells to halt the aging process (Baker et al., 2011; Chin et al., 2023). These observations suggest that the buildup of senescent cells during aging could fuel the advancement of aging and aging‐related illnesses. Consequently, there has been notable interest in developing drugs, termed “senolytics,” that target senescent cells to alleviate the progression of aging. Clinical trials for various potential senolytic drugs are already underway. However, these senolytic drugs primarily target several upregulated senescent cell antiapoptotic pathways (SCAPs), such as the p53‐mediated pathway and BLC‐2 family‐related pathway. Owing to the heterogeneous characteristics of senescent cells, they cannot completely clear the whole population of accumulated senescent cells (Chaib et al., 2022).

**FIGURE 1:**
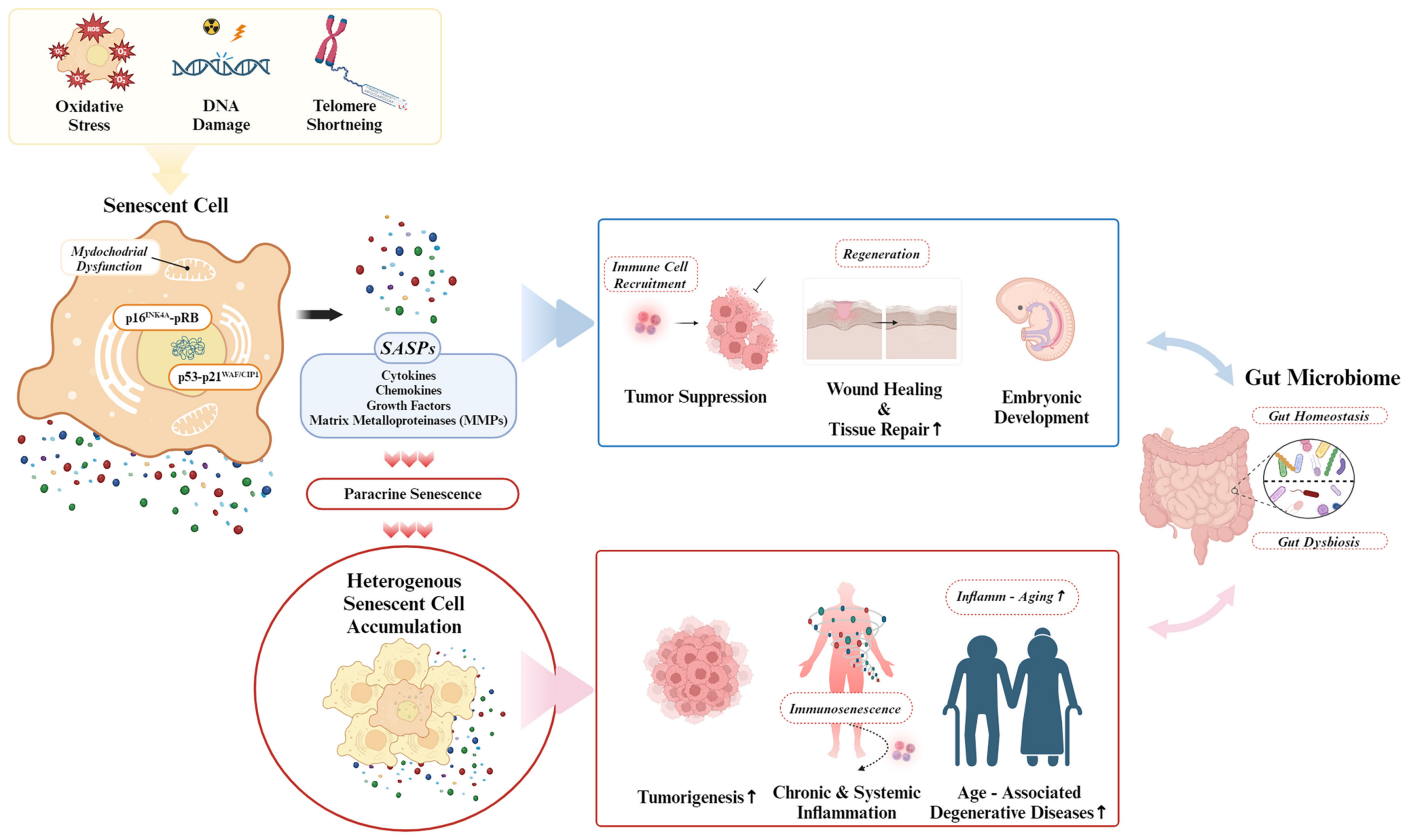
A summary of the multifaceted features of senescent cells and their impact on the gut microbiome. Cellular senescence arises from myriad stimuli, including DNA damage, telomere shortening, and oxidative stress, which disrupt normal cellular functions and induce cell cycle arrest through key pathways such as p16^INK4A^‐pRB and p53–p21^WAF1/CIP1^. While senescent cells play a crucial role in tumor suppression, wound healing, and embryonic development, they paradoxically exert harmful effects as the host ages. Immunosenescence impedes the clearance of senescent cells, leading to their propagation to neighboring cells through paracrine senescence. This complex interplay between stress‐induced factors and unique transcriptional responses during cellular senescence leads to the release of a variety of pro‐inflammatory SASPs, which contribute to a chronic inflammatory state, thereby accelerating the progression of Inflammageing. Also, accumulating evidence suggests that bidirectional interaction between the gut microbiome and senescent cells may cause gut dysbiosis, and conversely, gut dysbiosis may directly influence cellular senescence in gut‐resident cells. This interaction has significant implications for developing aging‐related diseases. However, little is known about the beneficial role of senescent cells in the intestine. Therefore, further research is needed to fully understand the interactions between cellular senescence and the gut microbiome during aging. (created with biorender.com).

Despite their negative impact, senescent cells also perform beneficial roles, including tumor suppression, by mobilizing the immune system through SASPs (Campisi & d'Adda di Fagagna, 2007; Feldser & Greider, 2007). Moreover, senescent cells have been implicated in tissue repair, defense against infections, and maintenance of tissue homeostasis (Demaria et al., 2014; Kita et al., 2022). Reports indicate that eliminating p16^INK4a^‐expressing cells in knock‐in mice can also harm health (Grosse et al., 2020). This duality suggests that indiscriminate elimination of all senescent cells could have adverse effects, advocating for strategies that target the harmful aspects of senescence while preserving its beneficial function (Amor et al., 2024; Chin et al., 2023; Smer‐Barreto et al., 2023). Developing senotherapeutic drugs that selectively inhibit SASPs or prevent the triggers of cellular senescence may offer a more nuanced approach to extending the health span and minimizing the side effects (Gasek et al., 2021; Zhang, Pitcher, et al., 2023) (Figure 1).

For these reasons, there is a need for large scale future efforts such as SenNet (Suryadevara et al., 2024), which should leverage bioinformatics tools, coupled with single‐cell transcriptomics, epigenomics, and proteomics, to decipher the heterogeneity of senescent cell populations and their dual effects on aging. Identifying markers to distinguish between beneficial and detrimental senescent cells is crucial for developing precise and personalized aging interventions. Such targeted approaches, which focus on the unique characteristics and effects of different senescent cell types, will enable the development of senotherapeutic strategies aimed at minimizing harm while maximizing benefits. This will be instrumental in contributing to healthier aging and the prevention of age‐associated diseases.

## 3 AGING‐ASSOCIATED CHANGES IN THE GUT MICROBIOME

The term “microbiome,” a blend of “microbiota” and “genome,” refers to the collective genetic material of microorganisms in a specific environment (Hou et al., 2022). In the human gut, a complex ecosystem of over 10^14^ microorganisms, comprising more than 2000 species that outnumber the human cell count, plays a crucial role in maintaining human health (Thursby & Juge, 2017). This gut microbiome is pivotal in digestion, nutrient absorption, immune function, and pathophysiological processes (Nagpal et al., 2018; Sbierski‐Kind et al., 2022; Shin et al., 2021; Sun et al., 2023). However, aging influences microbial diversity and composition, leading to microbiome dysbiosis (Clark et al., 2015; Jiao et al., 2021; Langille et al., 2014; You et al., 2022). In aged mice, this dysbiosis, coupled with increased gut permeability, facilitated the translocation of microbial products into the circulation, triggering systemic inflammation—a condition associated with low‐grade, persistent inflammation driven by senescent cells (e.g., increased levels of tumor necrosis factor (TNF) and IL‐6 in the blood) (Thevaranjan et al., 2017). This relationship underscores the role of the gut microbiome in aging and invites further exploration into its potential for promoting healthy aging.

As humans age, the microbiota, which produces short‐chain fatty acids (SCFAs), such as butyrate, diminishes. Butyrate, with its anti‐inflammatory properties, is produced through the bacterial fermentation of dietary fiber and resistant starch (Bach Knudsen et al., 2018; Canfora et al., 2015; Fernández et al., 2016; Wenzel et al., 2020). Notably, aging is associated with a decline in butyrate‐producing bacteria from the *Firmicutes* phylum, such as *Faecalibacterium prausnitzii*, *Eubacterium rectale*, *Clostridium septum*, and *Roseburia* species (Biagi et al., 2016). This reduction compromised gut integrity and immune function, which is further exacerbated by decreases in secondary bile acids and vitamins (Walrath et al., 2021). Concurrently, an increase in *Proteobacteria*, especially *Enterobacteriaceae*, raises gut lipopolysaccharide (LPS) levels, leading to cytotoxicity and immune response in intestinal cells (Mukhopadhya et al., 2012; Shin et al., 2015). Age‐related changes in the duodenal microbiome are influenced by disease status, medication use, and lifestyle factors, leading to increased *Proteobacteria* and decreased *Bacteroidetes*, affecting microbial diversity (Ghosh et al., 2022; Jackson et al., 2018). Certain genera like *Escherichia, Enterococcus*, and *Lactobacillus* increase with age, while *Klebsiella, Bilophila, and Clostridium* rise with medications and concurrent diseases (Leite et al., 2021). This dysbiosis and increased gut permeability facilitate the translocation of microbial components into the bloodstream, triggering systemic chronic inflammation and contributing to aging‐related diseases (DeJong et al., 2020; Fransen et al., 2017; Jackson et al., 2018).

Although extensive research has focused on gut bacteria, less is known about how gut fungi (mycobiome), viruses (virome), and protozoa change with age and contribute to age‐related diseases. This knowledge gap is due to the low proportion of fungi in the gut microbiome (less than 1%) and limitations in viral genome databases (Al Bataineh et al., 2020; Johansen et al., 2023; Wilmanski & Gibbons, 2023). Recent studies have started to address these gaps. For example, a study in China involving 251 participants aged 24–108 years, including 47 centenarians, found age‐related changes in the gut mycobiome (Pu et al., 2024). Long‐lived individuals had distinct fungal taxa, including *Penicillium* and *Aspergillus*, with an abundant *Candida* enterotype that correlated positively with *Bacteroides*, indicating that fungi‐bacteria interactions in aging. Similarly, a study of 389 Japanese participants, including 195 centenarians, revealed age‐related changes in the virome, with centenarians exhibiting distinct viral communities that interact with bacteria (Johansen et al., 2023). Centenarians had higher prevalence of *Crassvirales* and *Microviridae*, and viruses associated with *C. scindens*, along with reduced *Bacteroides* and *Faecalibacterium* viruses. Additionally, centenarians' gut microbiomes had higher sulfur metabolic activity, linked to viruses encoding sulfate‐reducing enzymes, enhancing mucosal integrity and pathogen resistance (Johansen et al., 2023). Further research is needed to understand how these changes relate to aging, age‐related diseases, and the gut mycobiome and virome (Balderramo et al., 2023; Cao et al., 2022; Iliev & Leonardi, 2017).

Despite critical insights, the intricate relationship between aging and microbiome alterations remains underexplored due to technological limitations. Advances in sequencing, metagenomic technology, and analytical tools are imperative for addressing individual variability and achieving precise analysis. Such advancements will enable the development of targeted interventions to mitigate the adverse effects of aging on the gut microbiome, promoting healthy aging (Bharti & Grimm, 2021; Ojala et al., 2023).

## 4 THE CORRELATION BETWEEN MUSCULOSKELETAL DISEASE AND GUT MICROBIOME DYSBIOSIS

The rapidly growing field of research into the relationship between gut dysbiosis and age‐related chronic disorders unveils significant connections with musculoskeletal conditions, which significantly impact older populations globally. This exploration delves into how these musculoskeletal conditions such as osteoporosis, sarcopenia, and knee osteoarthritis, are potentially influenced by the composition and health of the gut microbiome, underscoring the potential for targeted microbiome interventions to mitigate these conditions. While current research has established preliminary connections, future research is needed to further elucidate the molecular mechanisms involved and to develop effective therapeutic strategies.

*Osteoporosis*, affecting more than 200 million older people globally, is characterized by bone tissue deterioration and low bone mass, leading to fragile bones prone to fractures. The burgeoning field of microbiome research in bone health has expanded significantly, driven by understanding how metabolic pathways, the immune system, and hormonal balance influence bone metabolism, suggesting a potential role for the gut microbiome. Research has connected variations in the gut microbiota to the risk of osteoporosis, revealing higher levels of specific genera such as *Actinomyces*, *Eggerthella*, *Clostridium Cluster XVa*, and *Lactobacillus* in osteoporotic individuals compared to those with normal bone density (Das et al., 2019). Conversely, the reduced abundance of *Escherichia/Shigella* and *Veillonella* species in osteoporotic individuals compared to those with osteopenia (Das et al., 2019). Moreover, increased diversity and abundance of *Dialister* and *Faecalibacterium* have been observed in individuals with osteoporosis compared to those with normal bone density (Xu et al., 2020). KEGG pathway analysis revealed downregulated membrane transport and carbohydrate metabolism modules in osteoporotic individuals, which are essential for bone metabolism and cell viability. Increased levels of *Dialister* have also been linked to elevated levels of IL‐6, which induce bone loss. Chinese studies have also highlighted associations between the abundance of certain bacteria (*Bifidobacterium*, *Roseburia*, *Lactobacillus*, *Allisonella*, *Klebsiella*, and *Megasphaera*) and bone mineral density (BMD) levels (He et al., 2020; Li et al., 2019). In clinical trials, probiotic supplementation has shown promise in reducing bone loss in postmenopausal women, with one trial focusing on tibia BMD and another on lumbar spine (LS)‐BMD in Sweden (Jansson et al., 2019). Additionally, a Japanese trial found that *Bacillus subtilis* C‐3102 improved total hip BMD but had no significant effect on LS‐BMD (Takimoto et al., 2018). An Iranian study found that consuming a multispecies probiotic capsule daily for 6 months reduced TNF‐α levels, bone turnover‐related markers (collagen type 1 cross‐linked C‐telopeptide (CTX) and bone‐specific alkaline phosphatase (BALP)), parathyroid hormone (PTH) levels, suggesting that probiotics can suppress bone turnover and resorption, potentially improving bone health (Jafarnejad et al., 2017). However, further studies are required to confirm these mechanisms.

*Sarcopenia*, manifested by a decline in muscle mass and function (strength and performance) due to aging, is recognized as a prominent disease hindering physical activity by the aged population (Dennison et al., 2017). Though less explored than the gut–bone axis, the gut–muscle axis theory has recently underscored the significance of maintaining gut microbiome homeostasis in skeletal muscle metabolism and function (Liu et al., 2021). Studies in mice have demonstrated decreased skeletal muscle mass and function in germ‐free (GF) and antibiotic‐treated mice. Reintroducing gut microbiota from conventionally raised mice to GF mice increased skeletal muscle mass and reduced muscle atrophy markers. Treatment with SCFAs partly reversed skeletal muscle impairments in GF mice. In humans, specific microbial genera have been associated with higher lean mass and better physical performance in older adults (Cawthon et al., 2021; Song & Lee, 2020; Verschueren et al., 2013). For example, bacteria known for their anti‐inflammatory function and butyrate production, such as *Fusicatenibacter*, *Lachnospira*, *Roseburia*, *Eubacterium*, and *Lachnoclostridium* have declined, while *Lactobacillus* is more abundant in the fecal samples of pre‐sarcopenic and sarcopenic individuals (Kang et al., 2021). A large population‐based study in a Norwegian cohort (*n* = 5196, including both sexes) found increases in appendicular lean mass (ALM), a diagnostic factor of sarcopenia, and BMD correlated with three anabolic bacterial species: *Dorea longicatena*, *Coprococcus comes*, and *Eubacterium ventriosum* (Grahnemo et al., 2023). Preclinical studies revealed that the gut microbiome‐derived bile acid (BA)‐related pathway is significantly associated with muscle growth (Qiu et al., 2021, 2022). Primary BAs produced in the liver migrate to the ileum, where bile salt hydrolase (BSH) deconjugates them, converting them to secondary BAs. These BAs act as ligands for the farnesoid X receptor (FXR), promoting the secretion of fibroblast growth factor 15/19 (FGF15/19), which activates the ERK1/2 signaling pathway and increases muscle mass (Benoit et al., 2017). In aged mice, the relative abundance of *Firmicutes* increased, while *Proteobacteria* and *Bacteroidota* decreased. Strains with high BSH activity, like *Lachnospiraceae*, *Ruminococcaceae, Lactobacillaceae*, and *Bifidobacteriaceae*, decreased, leading to increased conjugated BAs like tauro‐beta‐muricholic acid (TβMCA), an FXR antagonist. This downregulates FXR‐FGF15/19 signaling, contributing to muscle loss (Benoit et al., 2017; Guo et al., 2021; Mancin et al., 2023; Qiu et al., 2021, 2022). Clinical trials are currently underway in Ireland to assess the effect of probiotic supplementation (*Bacillus coagulans*) on muscle protein synthesis in response to a plant‐based diet (Dublin & Group, 2019). Positive outcomes could provide a strategy to mitigate age‐related muscle loss and decline in physical function among older adults.

*Osteoarthritis* (*OA*), the most prevalent form of degenerative joint disease, is primarily characterized by the progressive loss of the cartilage matrix but also involves pathological changes in other joint components such as subchondral bone sclerosis, osteophyte formation, and synovial inflammation (Favazzo et al., 2020). Key risk factors for OA include systemic factors such as aging, obesity and local factors such as mechanical stress from excess weight and joint instability. These factors contribute to an increase in local and systemic inflammation (Berenbaum et al., 2017; Jeon et al., 2017, 2018), which is interconnected with the gut microbiome. A recent small‐scale study demonstrated worsening OA pathology when serum and synovial fluid contained high levels of bacterial LPS, accompanied by activated macrophages in the knee joint capsule and synovium (Huang et al., 2016). Additionally, the abundance of *Streptococcus* species was recently associated with increased knee joint inflammation and pain in non‐obese OA participants from a large population cohort study in the Netherlands (Boer et al., 2019). Considering the correlation between the metabolism of the gut microbiome and obesity (Huang & Kraus, 2016; Liu et al., 2019; Schott et al., 2018; Winer et al., 2016), gut dysbiosis‐derived metabolites (e.g., LPS) induce systemic inflammation by activating the macrophage and Toll‐like receptor (TLR) pathway in obesity, exacerbating OA‐related knee pain (Huang et al., 2016; Ohto et al., 2012). Human clinical trials have already shown that *Lactobacillus casei Shirota* and *Streptococcus thermophilus* have a beneficial effect on the management of knee OA (Lei et al., 2017; Lyu et al., 2020).

## 5 BURGEONING TIES: CELLULAR SENESCENCE AND GUT MICROBIAL SHIFT WITH AGING

The evolving understanding of gut microbiome alterations with age, especially in the context of cellular senescence and systemic inflammation, has profound implications for aging and health. This relationship suggests a bidirectional interaction where gut dysbiosis may exacerbate senescence and, conversely, senescent cells may influence microbiome's composition and function. This interaction has significant implications for developing aging‐related diseases, including musculoskeletal diseases, offering a potential target for therapeutic intervention. The accumulation of diverse senescent cell types, such as epithelial or immune cells in the intestine and their associated SASP with aging, could trigger changes in microbial diversity and microbiota‐derived metabolites within the gut environment. Conversely, metabolites produced by the gut microbiota can potentially influence cellular senescence in intestinal cells directly.

The intestinal epithelium, located near the luminal area housing the microbiota, comprises various subtypes of intestinal epithelial cells (IECs), including intestinal stem cells (ISCs), goblet cells, and Paneth cells (Hohman & Osborne, 2022). These cells serve a dual function as protective barriers, shielding host tissues from the luminal environment and as regulators of inflammatory balance within the gut. ISCs, found in the intestinal crypts, typically undergo self‐renewal via canonical WNT signaling, facilitating the replacement of damaged epithelial cells and maintaining the integrity of the intestinal barrier (Tian et al., 2015). However, the accumulation of senescent ISCs, alongside their persistent SASP secretion, creates an inflammatory environment, disrupting intestinal function and homeostasis (Funk et al., 2023). For example, exposure to iron radiation in mice resulted in increased ROS and ongoing DNA damage in ISCs, along with the accumulation of premature senescence and SASP markers (Kumar et al., 2019). Furthermore, aging contributes to diminished proliferation, self‐renewal, and motility of ISCs, exacerbating local inflammation and challenges in intestinal maintenance (Choi et al., 2018). These findings suggest that cellular senescence is significant in the intestinal epithelium and influences gut permeability and immune response, ultimately impacting overall gastrointestinal function.

Goblet cells produce the mucus layer, while Paneth cells secrete antimicrobial peptides such as α‐defensins and lysozyme to defend against bacterial invasion. Research indicates that the senescence of both cell types compromises intestinal barrier functions, manifested by reduced mucin production in goblet cells, diminished lysozyme secretion, and activation of Notum, a WNT inhibitor, in Paneth cells of aged mice (Elderman et al., 2017; Pentinmikko et al., 2019; Sovran et al., 2019). These disruptions in intestinal barrier integrity, caused by the senescence of both cell types, promote bacterial permeabilization and chronic inflammation (Branca et al., 2019). As a result, they can exacerbate systemic inflammation and compromise overall gut health.

In the lower portion of the epithelium layer, the lamina propria contains various immune cells (e.g., dendritic cells, macrophages, and B/T lymphocytes) that protect the host from pathogenic components translocated through the intestinal epithelial barrier by triggering an immune response (MacDonald, 2003; Montalban‐Arques et al., 2018). Recent research indicates that gut ileal germinal center (GC) B cells undergo cellular senescence in a bacteria‐dependent manner as individuals age. This highlights that age‐related intestinal barrier dysfunction, coupled with an increase in Gram‐negative bacteria, escalates microbiota permeabilization in ileal tissues, leading to prolonged stimulation of immune cells. Moreover, the senescence of germinal center B cells within Peyer's patches (PPs) and isolated lymphoid follicles (ILFs) diminishes IgA production and decreases diversity. This results in alterations in the binding affinity of IgA to gut bacteria, consequently impacting the gut microbiota composition. Importantly, in p16/p21‐ double knockout (DKO) mice, aging‐induced suppression of B cell proliferation seems to be alleviated, mitigating the reduction in IgA‐positive plasma cells and IgA clonotype diversity (Kawamoto et al., 2023; Kawamoto & Hara, 2024). However, in the context of other gut‐resident cells and the microbiota, the specific mechanisms by which the senescence of other resident cells directly influences changes in microbiome composition remain unclear. While the senescence of these cells may not directly alter the gut microbiome composition, it does impair the function of the intestinal barrier. This impairment can lead to increased gut permeability, contributing to gut dysbiosis, the deterioration of intestinal homeostasis, and the development of inflammatory diseases. These findings underscore the importance of regulating the senescent state of IECs and immune cells, particularly B cells, to promote healthy aging and maintain gut microbiome homeostasis. Further research is needed to elucidate the relationship between cellular senescence of other gut‐resident cells, increased gut permeability due to aging, and subsequent changes in the gut microbiome.

While it is well understood that senescent cells can play beneficial roles in various contexts, such as tumor suppression (Rodier & Campisi, 2011), embryonic development (Storer et al., 2013), wound healing (Demaria et al., 2014), and tissue repair (Chikenji et al., 2019) and regeneration (Paramos‐de‐Carvalho et al., 2021), little is known about their beneficial roles in the intestine. This is an area that requires further research to fully understand the potential positive effects of cellular senescence in the gut microbiome during aging.

The gut microbiome is crucial in metabolizing dietary nutrients to produce various bioactive metabolites, such as SCFAs, amino acids, and hormones through fermentation processes (Bernalier‐Donadille, 2010; Fernández et al., 2016; Oliphant & Allen‐Vercoe, 2019). Numerous studies have highlighted the effects of microbiota‐derived SCFAs on the intestinal environment. These effects include bolstering intestinal barrier integrity, increasing mucus production, modulating commensal microbes, and promoting immune tolerance, notably by maintaining the homeostasis of T regulatory cells (Tregs) (Chang et al., 2014; Corrêa‐Oliveira et al., 2016; Furusawa et al., 2013; Smith et al., 2013). On the other hand, a recent study demonstrates that butyrate, an SCFA released by *Porphyromonas* species, promotes colorectal cancer (CRC) development by inducing cellular senescence in the tumor environment (Okumura et al., 2021).

Secondary bile acids (BA), converted from primary BA via gut‐residing microbiota‐mediated fermentation, are also associated with maintaining intestinal homeostasis and barrier function, primarily due to their antimicrobial activity (Jin et al., 2024; Larabi et al., 2023). In a mouse study, several isoforms of lithocholic acid (LCA), including isoalloLCA and 3‐oxoLCA, a type of secondary BA, modulate the balance of Th17 and Treg cells differentiation within the intestinal lamina propria, thereby controlling host immune response through an anti‐inflammatory role (Hang et al., 2019). In line with this, an analysis of fecal samples from Japanese centenarians—those who are 100 years of age or older and are less vulnerable to age‐associated diseases—showed a rise in the strain of *Odoribacteraceae* that produces isoalloLCA. By exerting antimicrobial activities on gram‐positive bacteria such as *Enterococcus faecium* and *Clostridium difficile*, this preserves intestinal homeostasis (Sato et al., 2021). However, studies on obese mice reveal that deoxycholic acid (DCA), a type of secondary BA, circulates to the liver, where it causes cellular senescence of hepatic stellate cells (HSCs) because of DNA damage. In turn, it stimulates the secretion of SASP, which causes inflammation and accelerates the development of hepatocellular carcinoma (HCC) (Loo et al., 2017; Yamagishi et al., 2022; Yoshimoto et al., 2013). Depending on the type of microbial‐derived metabolites, diverse effects on cellular senescence can manifest in the tissue microenvironment. Also, the systemic circulation of these microbial products can profoundly influence human health by interacting with distal organ systems and regulating persistent, low‐grade systemic inflammation (Agus et al., 2021; Glowacki & Martens, 2020).

After heterochronic parabiosis between young and old mice, a significant reduction in systemic inflammation has been observed in old mice, suggesting that alleviating senescence‐related phenotypes in intestinal cells may enhance intestinal function and barrier integrity. This improvement is expected to solve problems related to microbial dysbiosis and systemic inflammation (Shin et al., 2021, 2022). Thus, further research is warranted to elucidate the specific metabolites responsible for these anti‐senescence effects and their mechanisms of action.

## 6 GUT MICROBIOME MODULATION FOR HEALTHY AGING

Modulating the complicated relationship between the gut microbiome and cellular senescence holds significant promise for sustaining gut microbiome equilibrium, which is pivotal for healthy aging. Strategies for microbiome modulation include biotics—probiotics, prebiotics, synbiotics, and postbiotics (Kim & Mills, 2024)—as well as fecal microbiota transplantation (FMT), and specific dietary regimens, such as caloric restriction (Ghosh et al., 2022; Nagpal et al., 2018; Vaiserman et al., 2017; Zhang et al., 2013).

*Probiotics* are live microorganisms that, when administered to the host, improve gut health by producing polyamines, SCFAs, and vitamins, and by promoting antimicrobial activity against pathogens. Well‐known probiotics like *Bifidobacterium* and *Lactobacillus* strains reduce age‐related inflammation (Boyajian et al., 2024). For example, *Bifidobacterium animalis subsp*. *lactis LKM512* enhances intestinal barrier function and reduces inflammatory markers, including TNF‐α, NF‐κB, and IL‐6, thus contributing to an extended lifespan by suppressing inflammageing (Matsumoto et al., 2011).

*Prebiotics* are substrates that promote the growth of beneficial bacteria like probiotics. Common prebiotics, such as carbohydrates and polyphenols, enhance the production of anti‐inflammatory metabolites like SCFAs, which strengthen epithelial defense mechanisms and reduce systemic inflammation (Diwan & Sharma, 2022; Martel et al., 2024; Roy & Dhaneshwar, 2023). For instance, the green tea catechin epigallocatechin gallate (EGCG) reduces DNA damage markers (e.g., p53, p21) and pro‐inflammatory cytokines secreted by senescent cells in mice, alleviating systemic inflammageing and improving gut microbiota (Sharma et al., 2022).

*Synbiotics* combine probiotics and prebiotics for synergistic effects on gut health. For example, the synbiotic mixture FCT (*Lactobacillus gasseri 505* and *Cudrania tricuspida* leaf extract in fermented milk) reduced pro‐inflammatory cytokines (e.g., TNF‐α, IFN‐γ, IL‐1β, and IL‐6) and increases anti‐inflammatory cytokines (e.g., lL‐4 and IL‐10) in a mouse model of CRC, demonstrating anti‐inflammatory and antioxidant activities (Oh et al., 2020). However, clinical use of probiotics and prebiotics is limited by the need to maintain cell viability and the modest health benefits of existing products (Roy & Dhaneshwar, 2023).

*Postbiotics* contain inactive microorganisms or their components, such as heat‐killed microorganisms and metabolites from probiotics (Kim & Mills, 2024). These interventions target inflammation or oxidative stress to attenuate inflammageing (Diwan & Sharma, 2022) and promote healthy aging by addressing cellular senescence (Chaib et al., 2022; Zhang et al., 2017; Zhu et al., 2021). Studies have demonstrated the anti‐inflammatory and antioxidant properties of metabolites from probiotics, including *Lactobacillus acidophilus*, *Lactobacillus casei*, *Lactococcus lactis*, *Lactobacillus reuteri*, and *Saccharomyces boulardi* in human colon epithelial cells (De Marco et al., 2018). In particular, the metabolites secreted by *Lactobacillus fermentum* have shown promise in alleviating senescence‐associated phenotypes, downregulating cell cycle inhibitors, such as p53‐ p21^WAF1^ and p16^INK4a^, inhibiting the development of SASP factors via NF‐κB transcription, and modulating the PI3K/Akt/mTOR pathway and AMPK signaling (R. Kumar et al., 2020) in a model of hydrogen peroxide‐induced senescence in 3T3‐L1 preadipocytes. Heat‐inactivated *Bifidobacterium adolescentis* has been shown to counteract colonic senescence‐linked alterations by bolstering intestinal integrity and stimulating the regeneration of Lgr5^+^ ISCs through the WNT signaling pathways in aging mice (Qi et al., 2023). These studies highlight the therapeutic potential of various biotics in combating cellular senescence and age‐related pathologies, possibly through modulating signaling pathways involved in senescence and inflammation.

FMT, a procedure involving the transfer of stool or stool‐derived microbiota from a healthy donor into a recipient, aims to address microbial dysbiosis and its associated diseases. Experiments have shown that transferring microbiota from young to aged mice can reverse age‐related alterations within the gut‐brain and gut‐retina axes in young recipients. Conversely, the microbiota from young mice has been shown to diminish the expression of inflammation‐associated cytokines (e.g., TNF‐α, IL‐6) in aged mice, highlighting microbial modulation can serve as an effective strategy against aging‐related inflammations and disease (Cheng & Fischer, 2017; Parker et al., 2022).

Furthermore, anti‐senescence agents, particularly senolytic drugs, have emerged as promising strategies for preventing inflammageing induced by microbial dysbiosis. The administration of a combination of senolytic drugs, dasatinib (D) and quercetin (Q), to mice has significantly reduced the senescent cell burden and the expression of SASP factors (e.g., TNF‐α, IL‐6, and CXCL1) in the intestine (Saccon et al., 2021). This suggests that clearing accumulated senescent cells can restore the microbial balance and inflammatory milieu, potentially improving health. Additionally, the natural flavonoid compound fisetin, targeting senescent cells, effectively reduces inflammation and restores beneficial gut bacteria, offering potential as a clinical treatment for inflammatory bowel diseases (IBDs) (Ashiqueali et al., 2024). Similarly, quercetin's ability to correct gut dysbiosis in rat models of bleomycin‐induced pulmonary fibrosis, particularly through the enhancement of *Akkermansia* abundance, underscores the interconnected roles of senolytics in rectifying dysbiosis and mitigating related diseases (Wu et al., 2023).

**FIGURE 2:**
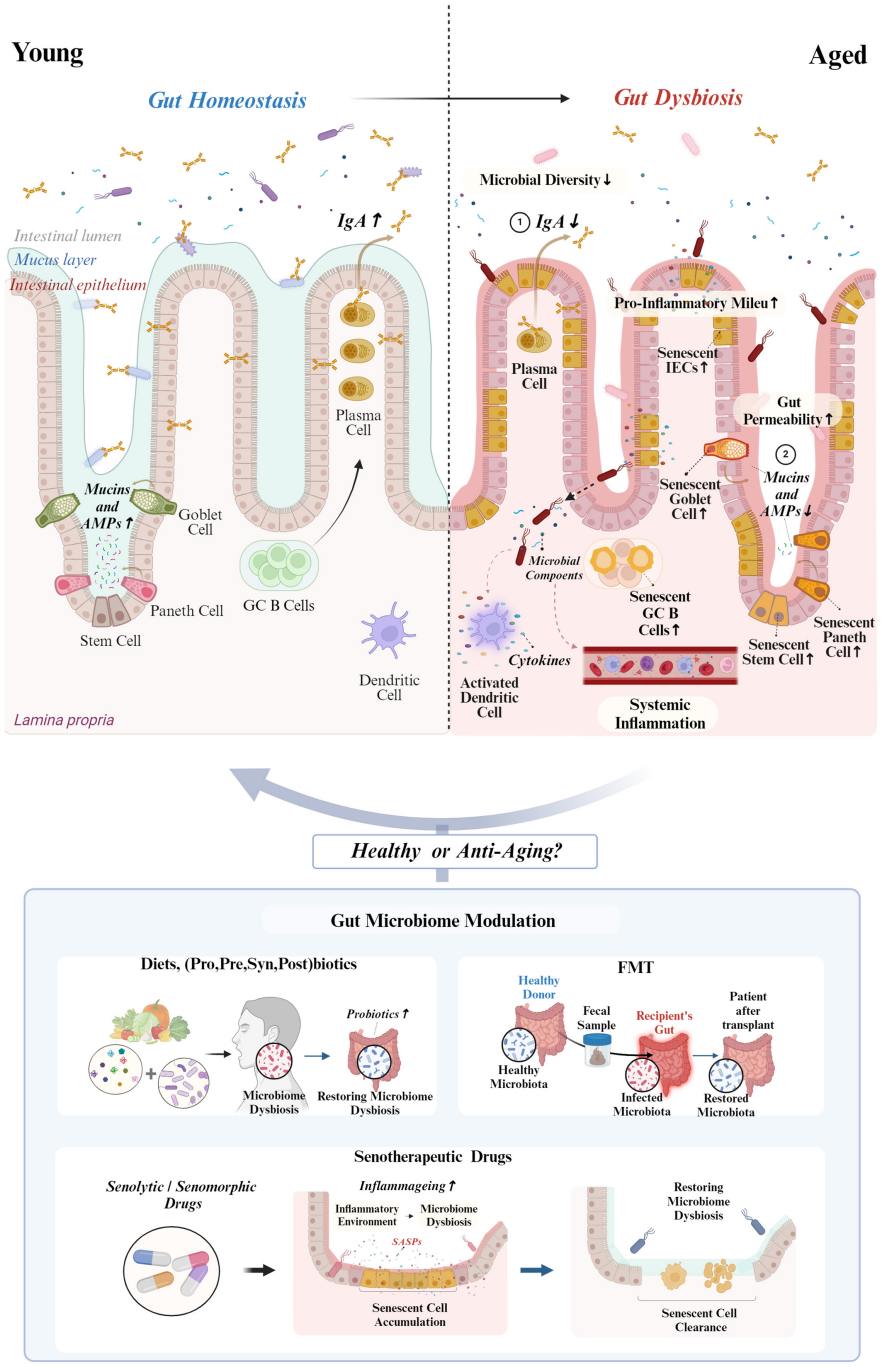
Gut microbiome alterations in aged people and strategies for gut microbiome modulation for healthy or anti‐aging. The aged intestinal gut microbiome presents a complex landscape marked by heightened gut permeability and inflammatory milieu. With age, various intestinal epithelial cells (IECs)—including goblet cells and Paneth cells—as well as intestinal stem cells (ISCs), undergo cellular senescence. This process impairs the regeneration of damaged epithelial cells and diminishes the production of mucin and antimicrobial peptides (AMPs), weakening the intestinal barrier. As a result, enhanced gut permeability allows for the infiltration of microbiota that induces cellular senescence in germinal center (GC) B cells. This, in turn, results in decreased IgA production and alterations in the composition of gut microbiota. The synergistic effect of senescence in immune and intestinal cells promotes gut dysbiosis and systemic inflammation, contributing to the development of age‐related diseases. Hence, endeavors to ameliorate age‐associated gut microbial dysbiosis focus on restoring host health through microbiome‐based interventions and targeting senescent cell therapies (senolytics or senomorphics), which is actively ongoing. (created with biorender.com).

These intervention strategies (Figure 2) underscore a dual approach towards achieving healthy or anti‐aging effects: (1) restoring microbiome dysbiosis to regulate cellular senescence and improve health, and (2) controlling cellular senescence to restore microbiome dysbiosis and achieve anti‐aging effects. The latter introduces a novel therapeutic strategy to enhance the symbiotic relationship between humans and their gut microbiota by modulating cellular senescence to restore health. As we have discussed, gut microbiome dysbiosis and cellular senescence interact bidirectionally, contributing to the deterioration of gut health with age. Depending on the circumstances, both factors must be targeted and modulated appropriately to maintain intestinal homeostasis. However, our understanding of the interaction between these two factors is still in its early stages. Therefore, it is crucial to study how cellular senescence and the gut microbiome interact to cause adverse outcomes during aging. This research will help identify new therapeutic targets for anti‐aging and healthy‐aging interventions.

## 7 CONCLUSION AND FUTURE PERSPECTIVES

The complex relationship between aging, cellular senescence, and gut microbiome alterations has been increasingly recognized as a pivotal factor contributing to systemic inflammation and physiological decline with aging (Diwan & Sharma, 2022; Kawamoto & Hara, 2024; Sharma, 2022). This recognition creates a novel therapeutic frontier for addressing aging‐dependent disorders, albeit with many barriers and limitations that remain to be addressed and considered (Chaib et al., 2022; Schafer et al., 2017; Soto‐Gamez & Demaria, 2017). First, understanding the interactions between the microbiome and cellular senescence is critical (Okumura et al., 2021). Future research should focus on delineating the mechanisms through which the gut microbiome influences cellular senescence and vice versa. This includes examining the role of SASP factors in modulating gut microbiome compositions and how microbial metabolites impact cellular aging.

Second, the development of senotherapeutic drugs, which aim to kill senescent cells or suppress their harmful effects selectively, respectively, should consider the influence of the gut microbiome on their efficacy and potential side effects. Understanding how these therapies affect the microbiome could lead to the optimization of treatment strategies to promote healthy aging. Modulating the gut microbiome through probiotics, prebiotics, and dietary interventions presents a promising avenue for mitigating cellular senescence, suggesting the need for identifying specific microbial strains or dietary components that can counteract senescence or its systemic impacts. Given the individual variability in microbiome composition, personalized microbiome interventions could become a cornerstone of future strategies, tailoring treatments to an individual's microbiome profile, genetic predispositions, and environmental factors (Ciernikova et al., 2023; Ghosh et al., 2022; Ma et al., 2023; Rafique et al., 2023).

Third, integrating the gut microbiome study with other aging theories, such as genomic instability, telomere attrition, and mitochondrial dysfunction, could offer understanding of aging, potentially revealing synergistic targets for interventions that simultaneously address multiple aging mechanisms. Yet, current interventions, such as FMT, face challenges related to individual variability and the potential transmission of pathogenic microbiota (Cheng & Fischer, 2017). The targeted modulation of specific senescent cells through senotherapeutic drugs tailored to an individual's unique characteristics emerges as a promising strategy for precision medicine (Niedernhofer & Robbins, 2018; Zhang et al., 2022; Zhang, Pitcher, et al., 2023).

Fourth, advances in metatranscriptomics allow for the detailed characterization of active microbial populations and their functional roles, while artificial intelligence (AI) enhances the development of senolytic drugs, offering hope for the precision targeting of senescence (Ojala et al., 2023; Smer‐Barreto et al., 2023). However, the complexity of aging process poses significant challenges for standardizing microbiome alteration patterns and pinpointing specific senescent cells and microbiota taxa using conventional computational methods (Ojala et al., 2023; Scepanovic et al., 2019). The advent of quantum computing introduces a transformative potential for the field, with its superior data processing speed, accuracy, and precision. Quantum computing is adept at precisely calculating drug interactions, binding energies, and chemical reactions, surpassing the capabilities of classical computing systems (Arute et al., 2019; Zinner et al., 2021). Moreover, it holds promise for modeling large‐scale simulations of protein interactions at the molecular level, a critical aspect for understanding the complex interplay between senescent cells and the microbiome (Gupta et al., 2023; Niraula et al., 2021). Leveraging quantum computing could revolutionize the selection of target microbiota and the development of senotherapeutic drugs aiming to restore microbiome homeostasis. As we get closer to these technological advancements, the future of aging research and therapy looks toward an era where modulating the gut microbiome and cellular senescence can be precisely tailored, offering new avenues for promoting healthy aging and mitigating the effects of aging‐related diseases (Okumura et al., 2021; Sato et al., 2021).

## AUTHOR CONTRIBUTIONS

We would like to express our gratitude to the organizers and participants of the A3 foresight meeting, which was focused on Cellular Senescence, for their valuable contributions to the discussion. D‐.H.J. conducted an extensive literature review and collaboratively prepared the initial draft of the manuscript. D‐.H.J., J‐.W.S., E.S., N.O., and O.H.J. wrote the original draft of the article with input from all co‐authors. DHJ prepared the figures. All co‐authors reviewed and edited the article.

## FUNDING INFORMATION

This research was financially supported by the National Research Foundation of Korea (NRF) grant funded by the Korean government (MSIT) (2020R1C1C1009921, RS2024‐00340798, and RS‐2023‐00220894).

## CONFLICT OF INTEREST STATEMENT

None declared.

## Data Availability

Data sharing is not applicable to this article as no new data were created or analyzed in this study.
